# Efficacy of Dietary Supplements on Sleep Quality and Daytime Function of Shift Workers: A Systematic Review and Meta-Analysis

**DOI:** 10.3389/fnut.2022.850417

**Published:** 2022-04-28

**Authors:** Yeqi Wu, Xueyan Huang, Congcong Zhong, Ting Wu, Dai Sun, Rui Wang, Qiang Zhan, Huasong Luo

**Affiliations:** ^1^Department of Massage, Hangzhou Hospital of Traditional Chinese Medicine Affiliated to Zhejiang Chinese Medical University, Hangzhou, China; ^2^Third Clinical Medical College, Zhejiang Chinese Medical University, Hangzhou, China; ^3^Department of Traditional Chinese Medicine Rehabilitation, Hangzhou Children's Hospital, Hangzhou, China

**Keywords:** shift work, dietary supplement, sleep quality, daytime function, meta-analysis

## Abstract

**Background:**

Dietary supplements (DSs) may be useful for managing shift work disorder. But the efficiency of outcomes in clinical trials using simulated shift work populations as subjects is controversial. This review explores the potential role of DSs for improving sleep quality, daily functioning, and mood among shift workers in the real world.

**Methods:**

A related literature search was conducted in PubMed, Web of Science, Embase, and Cochrane Library databases from their inception to July 2021. Information was collected on “shift work,” “irregular working hours,” “night shift,” “dietary supplements,” and “nutraceutical research data.” Sleep quality-related scales were the primary outcome measures. The meta-analysis was conducted using RevMan 5.4 (Cochrane Collaboration, London, England) and Stata 15.0 (StataCorp, LLC, College Station, TX, USA). Heterogeneity was examined by using *I*^2^ statistics, and publication bias was assessed via Egger's regression test.

**Results:**

Twelve studies, which involved 917 participants, met the inclusion criteria. The DS groups had significant improvement in sleep quality scores (8 randomized controlled trials [RCTs]: *p* = 0.04; standard mean difference (SMD), −0.45 [−0.88 to −0.03]) and daytime function (7 RCTs: *p* = 0.02; SMD, −0.50 [−0.92 to −0.08]). The DS groups did not have a significant improvement in psychomotor vigilance (4 RCTs: *p* = 0.25; SMD, 0.52 [−0.36 to 1.41]), depression (5 RCTs: *p* = 0.14; SMD, −0.19 [−0.45 to 0.06]), or anxiety (4 RCTs: *p* = 0.27; SMD, −0.23 [−0.65 to 0.18]). All RCTs suggested a positive safety profile for DSs.

**Conclusions:**

The findings of this meta-analysis indicated DSs may be beneficial for improving sleep quality and daytime function in shift workers. Although there is a wide range of DSs, the small amount of literature included for each type does not allow for subgroup analysis to be used to eliminate high heterogeneity. We have not yet included literatures on other languages either. Given these limitations of the study, there is still a need for more well-designed randomized controlled trials so that our review can be updated in the future to make the results more conclusive.

**Systematic Review Registration:**

https://www.crd.york.ac.uk/prospero/display_record.php?RecordID=273558, PROSPERO: CRD42021273558.

## Introduction

Shift work refers to working time arrangements that differ significantly from society's traditional working hours (e.g., 9 a.m.−5 p.m.), or even night work that is completely reversed (1). It cannot be ignored that disruption of circadian rhythms caused by the inability to adapt to shift work may be a major trigger of sleep disturbances (2), cardiovascular disease (3), stroke (4), metabolic syndrome (5), mood problems (6), cancer (7), and sudden death (8). Furthermore, shift work negatively impacts job performance (9), driving safety (10), and health outcomes during pregnancy (11). The best way to avoid shift work disorders altogether is probably to eliminate shift changes. However, owing to the pressures of life, only a small percentage of people can do this. Thus, identifying effective, safe, and acceptable prevention and treatment modalities to maintain health in the long term for shift workers is important.

The prevention and treatment of shift work disorder is mostly a stepwise approach, which is generally conducted in a parallel and multimodal manner. This approach includes pharmacological interventions such as sedative-hypnotics, wake promoters, and dietary supplements (DSs) (12) and includes non-pharmacological therapies such as bright light, napping, physical exercise, and sleep education (13). DSs have been widely available for people with sleep disturbances because of their additional benefits of being more affordable and easier to implement (14), but their therapeutic potential in the field of shift work disorder (SWD) remains to be determined.

In addition, questions concerning the applicability of measurements obtained after simulated shifts of healthy people to the real world prompted this study. Excessive stress due to work and social relationships in real life can lead to a vicious cycle of circadian rhythm disruption (15). Therefore, in our review, we deliberately excluded trials with simulated shifts to better reflect the clinical efficacy of DSs in the real world. The aim of this review was to assess the efficacy and safety of DSs for shift workers.

## Materials and Methods

This review followed the Preferred Reporting Items for Systematic Reviews and Meta-Analyses (PRISMA) guidelines (Supplementary File 1) (16). This prospective meta-analysis is registered in the International Prospective Register of Systematic Reviews (PROSPERO) and the registration number is CRD42021273558. This review had no direct patient or public involvement.

### Eligibility Criteria

All randomized controlled trials (RCTs) (including crossover studies) were included that involved humans participating in shift work and compared the clinical efficacy of at least one dietary supplement in any form with placebos. The duration and frequency of therapy were not limited. We did not consider age, sex, education, ethnicity, and occupation of the participants. The primary outcomes were changes in the score of the Pittsburgh Sleep Quality Index, Insomnia Severity Index, or other scales used to assess sleep quality. The secondary outcomes were daytime function, as assessed by the Epworth Sleepiness Scale, Karolinska Sleep Scale, or Chalder's Fatigue Survey. We also focused on psychomotor vigilance and mood outcomes, which were evaluated by reaction time and a self-assessment scale, respectively. However, a literature report was excluded if it included participants who experienced serious underlying diseases or cognitive-communication disorders, were pregnant, or were healthy and had undergone simulated night work. If more than one RCT was carried out with the same population, the most comprehensive one was included in the review.

### Literature Search

The following electronic databases were searched from their inception to July 31, 2021: PubMed, Web of Science, Embase, and Cochrane Library. Furthermore, the reference lists of the included literature, previous relevant reviews, and clinical trial registries were manually searched to find additional trials that met our inclusion criteria. The search was limited to English-language papers with no other restrictions applied. Exhaustive search strategies for each electronic database were developed by the review team members. The search strategy contained relevant terms, based on (1) “shift work” or “irregular working hours” or “nightshift” AND (2) “dietary supplements” or “nutraceutical” AND (3) “randomized clinical trial.” The search tactics are shown in Supplementary Files 2–5.

### Selection Criteria

The search results extracted from the aforementioned electronic databases and additional trials were managed through Endnote X9 (Clarivate, London, UK). Two reviewers (Y. Wu and C. Zhong) proceeded independently in a three-step sequence: removing duplicate literature, reviewing the titles and abstracts, and acquiring the full text for screening to exclude articles, based on the eligibility criteria. Any disagreements were discussed and resolved in discussion with a third reviewer (R. Wang).

### Data Extraction

Two investigators (X. Huang and D. Sun) independently extracted information from the selected articles, which were collected and sorted by a predefined extraction template. The extracted information included general information (e.g., reference identification, first author, country, publication year, and sample size); characteristics of the patients (e.g., age, sex, and comorbidity); characteristics of the shift work (e.g., occupation and shift schedule); methodology of the trial (including but not limited to randomization, allocation concealment, and blinding and its methods); characteristics of interventions; measurement of outcomes; adverse events; follow-up period; and funding. We attempted to contact the corresponding authors of the studies via email addresses to request missing data. The extracted information was cross-checked by X. Huang and D. Sun. For disagreements, mediation was conducted by a third investigator (R. Wang).

### Risk of Bias

Two researchers (Y. Wu and X. Huang) independently assessed the quality of the selected studies, using the Cochrane Collaboration's tool for RCTs (17). Each domain was determined to be a low risk of bias, unclear bias, or high risk of bias by using Review Manager 5.4 (RevMan; Cochrane Collaboration, London, England; available at https://training.cochrane.org/online-learning/core-software-cochrane-reviews/revman). Any disagreement was resolved by discussion or with the help of the third author (R. Wang).

### Statistical Analyses

Continuity correction was used for cells with zero values. A *p*-value <0.05 was considered statistically significant, unless otherwise specified. The continuous data included sleep quality, daytime function, psychomotor vigilance, depression, and anxiety, which were measured differently. The standard mean difference and 95% CI were used for effective evaluation. To ensure uniformity of the included data, all data were used in the intent-to-treat analysis. The traditional pair-wise efficacy data in populations were synthesized and statistically analyzed in Review Manager 5.4 by random effects models (weighted by the inverse of the variance). Given that a few studies did not report changes from the baseline data, we selected post-treatment data for the comparison of symptoms. If the mean or standard deviation was missing and the data were still not available after contacting the original author, we unfortunately had to exclude the data.

### Assessment of Heterogeneity and Stability

Between-study heterogeneity was assessed using the χ^2^ test and *I*^2^ test. Based on the *Cochrane Handbook*, the *I*^2^ was considered “substantial” if its value was >60%; “moderate,” 30–60%; and “non-important,” <30% (18). An *I*^2^ statistical value of 50% or higher indicated the presence of heterogeneity. To further assess the influence of heterogeneity on the conclusions of the meta-analysis, meta-regression and subgroup analysis were conducted to assess the primary outcome data, based on the differences in sample size, year of publication, country, DS type (i.e., melatonin and non-melatonin), occupation, and study quality (depending on whether random sequence generation or allocation concealment were used to determine the quality [i.e., high or low] of the studies). A sensitivity analysis was conducted in each domain of the primary outcome to assess the stability of the meta-analysis. Egger's regression test, using STATA 15.0 (StataCorp LLC., College Station, TX, USA), was also used to statistically assess publication bias.

## Results

### Characteristics of Included Studies

**Figure 1:**
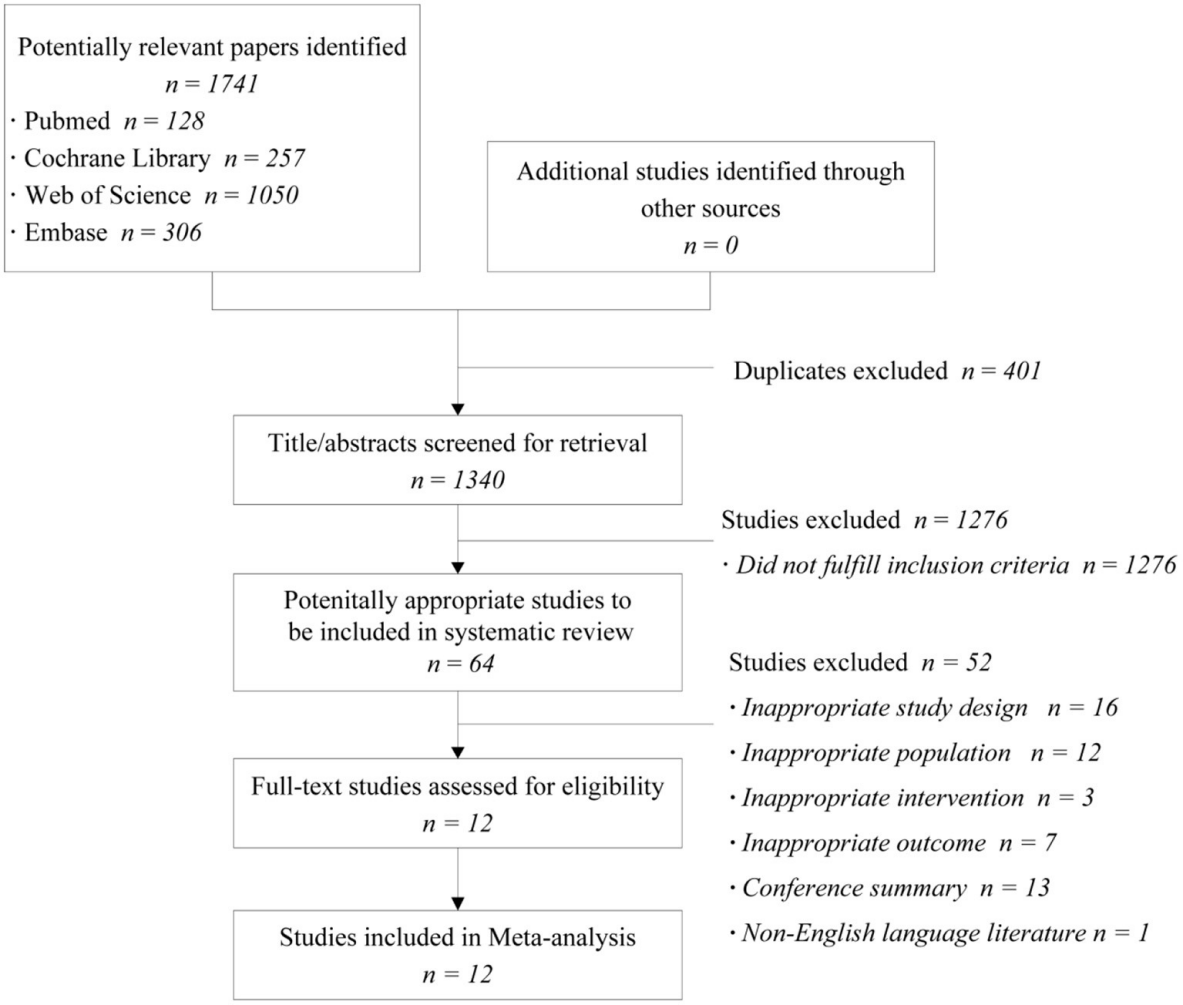
Flow diagram of the study selection process.

The search strategy retrieved 1,340 relevant articles, after removing duplicates. After a title/abstract review, the full texts of 64 potentially eligible articles were reviewed (Figure 1). Of these, only 12 studies (19–30) met the inclusion criteria, and the studies' characteristics are summarized in Table 1. One study (26) had four comparative groups; one study (29) had three comparative groups, and the remaining 10 studies had two comparative groups. All trials were conducted in non-simulated environments. Most participants were engaged in medical-related work, whereas other participants were factory workers or police officers. One trial (29) did not distinguish in detail between occupations. The included trials involved 917 participants, and the sample sizes ranged from 15 participants to 172 participants. Six of these studies (20–24, 28) were crossover trials in which the intervention was melatonin. The interventions used in the remaining six studies were probiotics (27, 29), zinc preparations (19), caffeine (25), omega 3/vitamin C (26), and herbs (30).

**Table 1:** Characteristics of the included studies.

**Author, year**	**Study design**	**Setting**	**Occupation**	**Intervention, group *N***	**Control, group *N***	**Outcomes measure**				**Adverse events**
						**Sleep**	**Daytime function**	**Psychomotor vigilance**	**Mood**	
Baradari et al. (19)	RCT, 2 arms	Iran	ICU nurses	Zinc sulfate, 27	Placebo, 26	PSQI	/	/	/	None
Bjorvatn et al. (20)	RCT, 2 arms, crossover	Norway	Oil well workers	Melatonin, 16	Placebo, 16	SQS	KSS	Reaction-time test (missing)	HADRS	NR
Cavallo et al. (21)	RCT, 2 arms, crossover	The United States	Pediatric residents	Melatonin, 35	Placebo, 38	SQS	/	Reaction-time test	POMS	All events were mild in severity and not statistically significantly different between the groups.
Farahmand et al. (22)	RCT, 2 arms, crossover	Iran	Emergency medicine residents	Melatonin, 48	Placebo, 48	/	KSS	/	POMS	All events were mild in severity and not statistically significantly different between the groups.
Folkard et al. (23)	RCT, 2 arms, crossover	The United Kingdom	Police officers	Melatonin, 7	Placebo, 8	SQS	/	Reaction-time test	Mood scores (missing)	NR
Sadeghniiat-Haghighi et al. (24)	RCT, 2 arms, crossover	Iran	Nurses	Melatonin, 86	Placebo, 86	SQS	/	/	/	None
Huffmyer et al. (25)	RCT, 2 arms	The United States	Anesthesiology residents	Caffeine, 13	Placebo, 13	/	ESS	/	/	NR
Khajehnasiri et al. (26)	RCT, 4 arms	Iran	Oil refinery workers	Vitamin C, 12	Placebo, 34	/	/	/	BDI	NR
Khajehnasiri et al. (26)				Omega 3, 11	Placebo, 34					
Khajehnasiri et al. (26)				Omega 3 + Vitamin C, 11	Placebo, 34					
Smith-Ryan et al. (27)	RCT, 2 arms	The United States	Health care employees	Probiotic, 15	Placebo, 18	/	/	CFS	HADRS	None
Thottakam et al. (28)	RCT, 2 arms, crossover	The United Kingdom	Acute care nurses and trainee doctors	Melatonin, 25	Placebo, 25	VSH sleep scale	ESS	Reaction-time test	/	All events were mild in severity and not statistically significantly different between the groups.
West et al. (29)	RCT, 3 arms	Australia	Medical staff, transport, services and security workers	Probiotic_a, 29	Placebo, 15	PSQI	/	/	/	NR
West et al. (29)				Probiotic_b, 29	Placebo, 14					
Zhang et al. (30)	RCT, 2 arms	China	Nurses	Herbs, 23	Placebo, 15	ISI	/	Reaction-time test	HADRS	None

*RCT, randomized controlled trial; ICU, intensive care unit; PSQI, Pittsburgh Sleep Quality Index; SQS, sleep quality scores; KSS, Karolinska Sleep Scale; HADRS, Hospital Anxiety and Depression Scale; NR, not reported; POMS, Profile of Mood States; ESS, Epworth Sleepiness Scale; CFS, Chalder's Fatigue Survey; VSH, Verran and Snyder-Halpern; ISI, Insomnia Severity Index*.

### Risk of Bias of Included Studies

**Figure 2:**
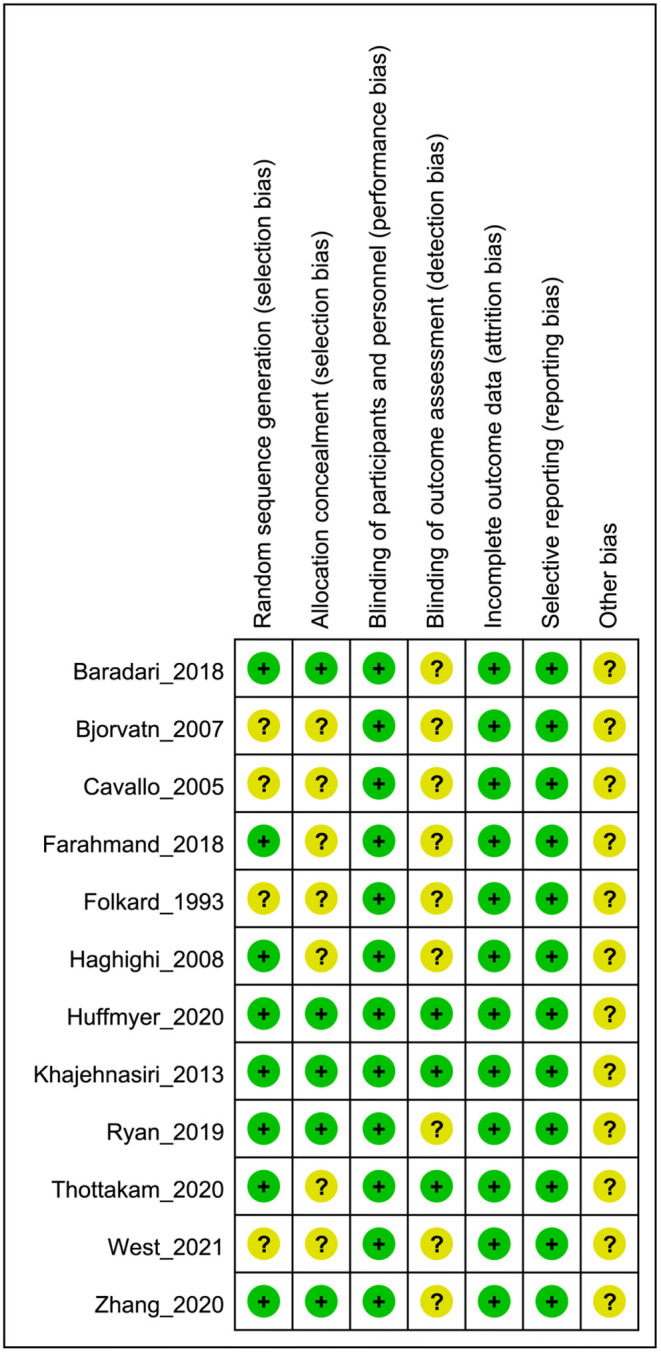
Risk of bias summary.

The risk of bias summary for each included trial is shown in Figure 2. Five studies (19, 25–27, 30) had a low risk of bias because the generation of random sequences and the allocation concealment were adequate. However, the description of randomization and allocation was unclear in four studies (20, 21, 23, 29), and the remaining three studies (22, 24, 28) described only the method of random scheme generation. All studies were conducted in a double-blind, placebo-controlled trial design. Only three studies (25, 26, 28) noted blinding of the outcome assessment, and in contrast, the other studies (19–24, 27, 29, 30) were unclear. In all trials, the quality of incomplete outcome data was adequate and no selective reporting of outcomes was observed. Other biases were classified as “unclear.”

### Quantitative Synthesis

#### Sleep Quality

**Figure 3:**
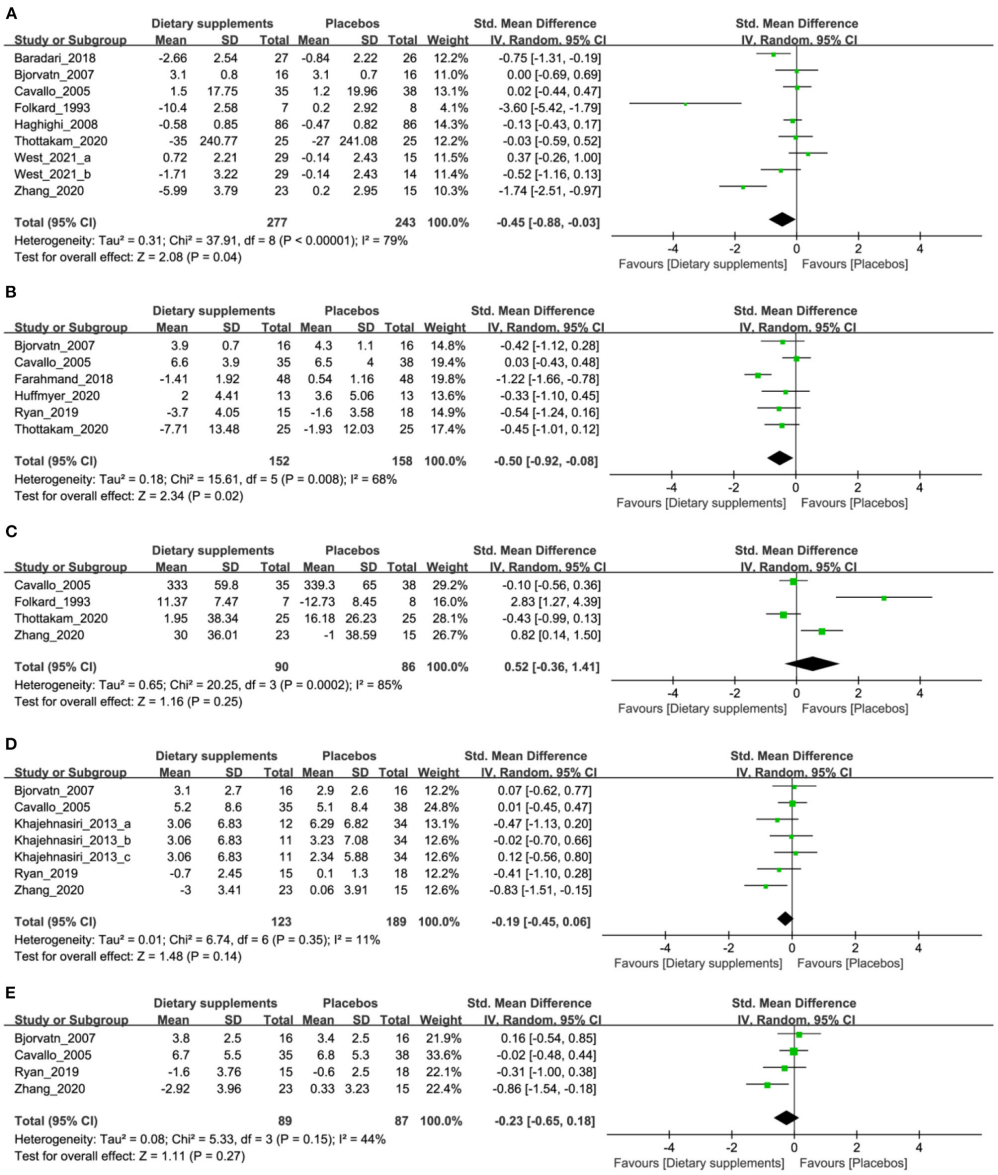
The forest plots illustrate the meta-analysis findings of the outcomes of dietary supplements (DSs) vs. those of the placebos for shift workers. The outcomes analyzed were **(A)** sleep quality, **(B)** daytime function, **(C)** psychomotor vigilance, **(D)** depression, and **(E)** anxiety.

This outcome indicator was assessed in 8 RCTs, 9 arms, and 520 participants. Two hundred seventy-seven participants taking DSs had a significant improvement in sleep quality scores, compared with the participants (*n* = 243) taking placebos (random effect [RE]: standard mean difference [SMD], −0.45; 95% CI, −0.88 to −0.03, *p* = 0.04; Figure 3A), but heterogeneity was evident (*I*^2^ = 79%, *p* < 0.00001).

#### Daytime Function

Seven RCTs, which involved 310 participants, reported changes in daytime function. In this cohort, 152 participants received DSs and 158 participants received placebos in the control groups. Heterogeneity was evident (*I*^2^ = 68%, *p* = 0.008). An analysis showed that DSs could improve daytime function (RE: SMD, −0.50; 95% CI, −0.92 to −0.08, *p* = 0.02; Figure 3B).

#### Psychomotor Vigilance

Four studies involving 176 participants were included in our meta-analysis of the change of psychomotor vigilance. The DS groups did not demonstrate a significant improvement in reaction times when compared with the placebo groups (RE: SMD, 0.52; 95% CI, −0.36 to 1.41, *p* = 0.25; Figure 3C).

#### Depression and Anxiety

Five RCTs (representing 7 arms) reported data on depression and four trials (representing 4 arms) reported data on anxiety. Heterogeneity was not significant in studies that compared depression scores (*I*^2^ = 11%, *p* = 0.35), but heterogeneity was significant in studies assessing anxiety (*I*^2^ = 44%, *p* < 0.15). This data analysis showed that DSs could not improve depression (RE: SMD, −0.19; 95% CI, −0.45 to 0.06, *p* = 0.14) and anxiety (RE: SMD, −0.23; 95% CI, −0.65 to 0.18, *p* = 0.27; Figures 3D,E).

#### Safety Profile

Adverse reactions were recorded in three of the 12 studies (21, 22, 28). However, no statistical difference existed in the incidences of adverse events possibly related to the treatment recorded for the DS groups, compared with the placebo groups. Four other studies (19, 24, 27, 30) even highlight the absence of adverse events. All of these results suggested a positive safety profile for DSs.

### Meta-Regression

A meta-regression was conducted to determine whether the observed efficacy on sleep quality was similar between different years of publication, DS types, occupations, and study quality. A significant association was found between sleep quality and the factors of years of publication, occupations, and study quality (Table 2).

**Table 2:** Meta-regression.

**Variables**	**Coefficient**	**Standard error**	***p***	**95% CI**
Sample size	−0.019	0.011	0.092	−0.040 to 0.003
Year of publication	0.194	0.066	0.004	0.064 to 0.324
Countries	−0.384	0.220	0.081	−0.816 to 0.048
DS types	1.602	0.831	0.054	−0.026 to 3.230
Occupations	−1.170	0.463	0.012	−2.077 to −0.262
Study quality	9.517	3.472	0.006	2.713 to 16.321

*CI, confidence interval; DS, dietary supplement*.

### Subgroup Analysis

Although the meta-regression revealed three potential factors contributing to heterogeneity, our attempts to further distinguish subgroups of shift workers across publication years and occupations were abandoned, as most studies of publication years did not find a pattern of grouping and their definitions of occupation were vague. Ultimately we selected different DS types and study quality for the subgroup analysis, based on the clinical characteristics of the included studies. The results showed that, compared with lower quality studies, the higher quality studies indicated a superior improvement in sleep quality for participants using DSs. However, we did not find better efficacy for DSs within the melatonin or the non-melatonin subgroups (Table 3 and Supplementary File 6).

**Table 3:** Subgroup analysis.

**Variables**	**Eligible studies *N***	**Eligible arms *N***	**Frequency**		**SMD (95% CI)**	***p***	**Heterogeneity Test**		**Effect model**
			**Intervention, group *N***	**Control, group *N***			***p***	***I*^2^, %**	
Different quality of studies									Random
High quality	2	2	50	41	−1.21 (−2.17 to −0.24)	0.01	0.04	76%	
Low quality	6	7	227	202	−0.65 (−1.02 to −0.27)	0.35	0.005	79%	
Different types of DS									Random
Melatonin	5	5	169	173	−0.25 (−0.74 to 0.24)	0.31	0.005	73%	
Other types	3	4	108	70	−0.64 (−1.43 to 0.15)	0.11	0.0005	83%	

*DS, dietary supplement*.

### Sensitivity Analysis

**Figure 4:**
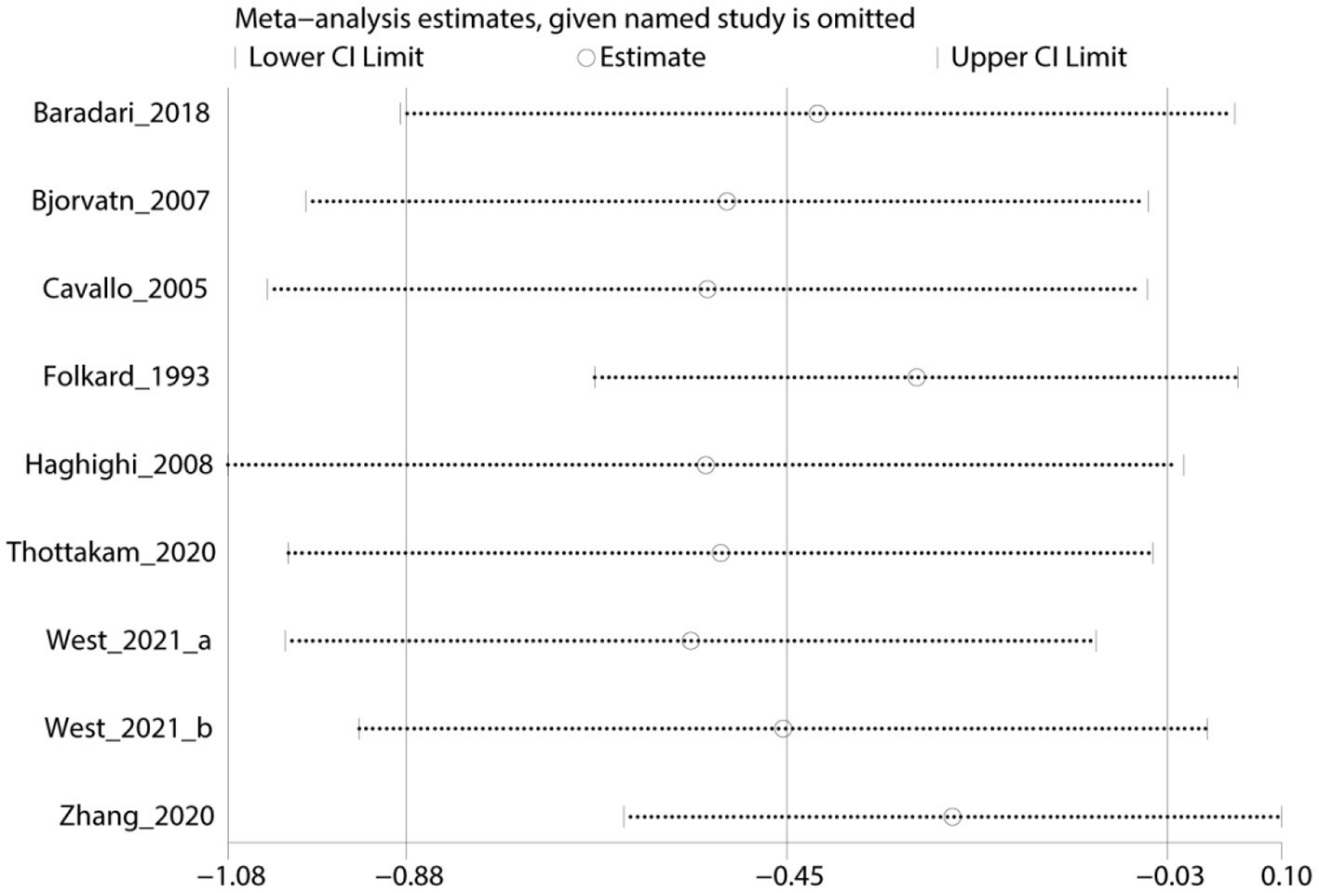
The forest plot illustrates the sensitivity analysis findings for the primary outcome of sleep quality (one RCT is omitted). CI, confidence interval; RCT, randomized controlled trial.

A sensitivity analysis was conducted with the results of the primary outcome indicator sleep quality to assess the stability of the meta-analysis. When any single study was deleted, the corresponding pooled SMD remained at a 95% CI of −0.88 to −0.03, which suggested that the meta-analysis was stable (Figure 4). However, the results from RevMan showed that the statistical significance of the differences changed after the deletion of some studies, possibly because of differences in study quality or sample size between these and other trials (Supplementary File 7).

### Publication Bias

**Figure 5:**
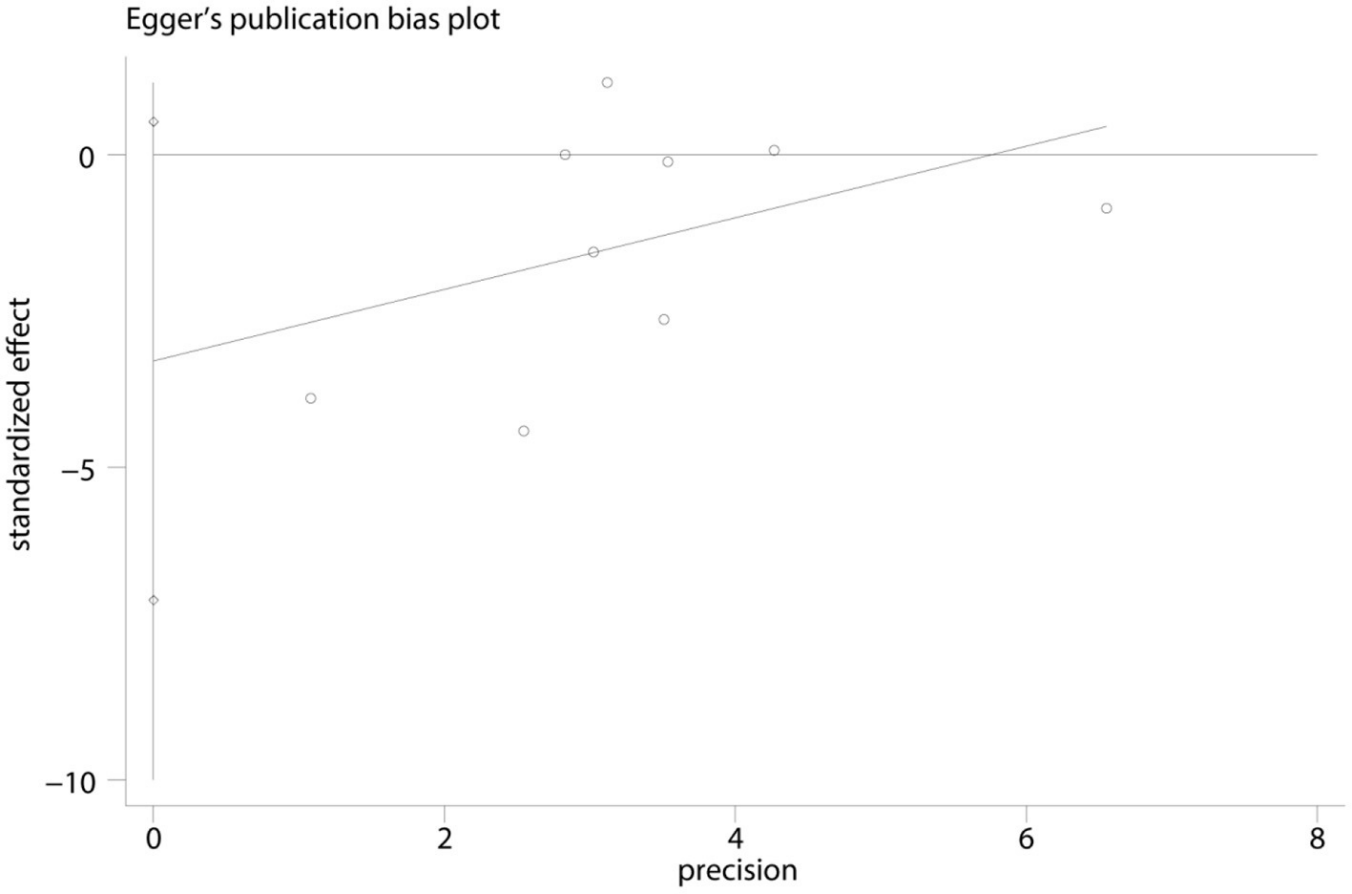
Egger's plot on sleep quality for the evaluation of the publication bias of the literature.

We found no evidence of publication bias for DS intervention when using Egger's regression test (*t* = −2.04; 95% CI, −7.13 to 0.53; *p* = 0.081; Figure 5).

## Discussion

The purpose of this review was to explore the potential role of DSs in improving sleep quality, daily functioning, and mood among shift workers. The key findings of this systematic review and meta-analysis indicated that, compared with placebos, DSs demonstrated a statistically significant difference in improving sleep quality and daytime function for participants. However, the change in psychomotor vigilance and mood was not significant. Perhaps the most important finding was that although a limited number of studies reported the presence of adverse events such as sleepiness, dizziness, and headache, all adverse effects were mild and transient, and the differences between the DS and the control groups were not statistically significant. No participants withdrew from the studies because of adverse events. In subgroup analyses, high-quality studies showed that DSs had a clear clinical effect, but low-quality studies showed no difference. It is interesting to consider that there was no difference in the efficacy of DS in either the melatonin or non-melatonin subgroups, this change in outcome may be related to the reduced sample size of the subgroups.

To the best of our knowledge, this study is the first systematic review and meta-analysis to examine the effects of DSs on SWD in real working environments. In a recent systematic review (12) of pharmacological interventions for SWD, the clinical efficacy of melatonin and that of caffeine were assessed. The findings suggested that melatonin may improve sleep length after night shifts, but may not improve other sleep quality parameters, whereas pre-shift caffeine increased alertness during the night shift. Investigators in another review (31) similarly concluded that caffeine may be an effective intervention for improving performance in shift workers. However, both studies lacked high-quality evidence and did not involve other types of dietary supplements. Consistent with these reviews, we reiterated the positive impact of DSs on sleep quality and work performance among shift work populations. SWDs are often accompanied by changes in mood (32); therefore, in our study, we evaluated the effect of DSs on the emotional state. However, we did not find that DSs had a better effect on mood in SWD than did placebos.

Uncertainty about the action mechanism of DSs may be the primary cause contributing to their limited promotion because they affect sleep quality, endogenous melatonin metabolism, positive regulation of the sleep–wake cycle, and alterations in inflammatory conditions closely associated with insomnia (33). For example, probiotics are believed to bring health benefits to patients with insomnia through the activity of the gut–brain axis (34). The mechanisms regarding the role of vitamins and herbs in sleep are similar and are probably associated with anti-inflammatory and antioxidant activity (35, 36). Of note, this area of research has received increasing attention because links between SWD and many major diseases of modern society have been established (32).

Compared with other intervention options, safety and adherence are also issues to consider when choosing DSs. Pharmacological hypnotics and wake-promoting drugs have a low safety profile and significant adverse effects; thus, the ingestion of drugs to manage SWD may not meet with widespread acceptance (37). Light therapy has been used effectively in SWD, but the time needed for bright light treatment may conflict with work tasks, and light exposure in the surrounding environment may be difficult to control. The application of laboratory-based knowledge to the real world can be problematic (2, 13). Our results suggested that consumption of DSs was comparatively safe with no serious adverse events. Researchers in a previous systematic review (38) on the safety of DSs drew a similar conclusion and proposed that the evaluation of DSs use and their safety should be regularly included in clinical trials. In addition, most of the studies we included did not address follow-up; therefore, the long-term efficacy of DSs has yet to be determined. Future research is urged to incorporate adequate follow-up with a long-term duration.

Despite the partially encouraging results of our study, several limitations should be noted. First, heterogeneity existed in the main outcome indicators, and through a meta-regression, we identified the year of publication, occupation, and study quality as possible sources. An attempt to analyze subgroups by year of publication and occupation unfortunately failed, as no grouping pattern was found for them. We were only able to select the types of DSs and study quality for subgroup analysis based on clinical characteristics, but there was still some heterogeneity. The findings were also interesting in the results of the sensitivity analysis. The outcome of sleep quality remained stable, as demonstrated in Figure 4, although we found that the statistical significance of the differences changed after removing several studies (19, 23, 29, 30), possibly because of differences in the study quality or sample size of these and other trials. Second, despite our efforts to conduct a comprehensive search of the literature, some relevant studies may have been missed because they were in languages other than English, which limited the amount of the included literature. Our systematic review did not include studies in Chinese, even though herbs are very popular in China. As the quality of clinical studies published in Chinese improves, future meta-analyses may include more trials using Chinese herbal medicine for SWD. Finally, populations in work-shift situations need prolonged attention. However, the included studies lacked follow-up information, thereby resulting in a lack of basis for evaluating the long-term efficacy of DSs.

## Conclusion

Our meta-analysis showed that DSs improved sleep quality and daytime function in shift workers, but they had no significant effect on psychomotor vigilance and mood. Thus, DSs could potentially be valuable clinically in improving sleep quality and daytime function in shift workers. Unfortunately, the small amount of the literatures included for each type of dietary supplement does not allow us to know how the different DSs compare. More high quality multi-arm and crossover designed RCTs are needed in the future to enable us to update the findings of this meta-analysis in time and provide the best options for dietary supplement management for shift workers.

## Data Availability Statement

The original contributions presented in the study are included in the article/Supplementary Material, further inquiries can be directed to the corresponding author/s.

## Author Contributions

YW and CZ: writing—original draft. YW, XH, CZ, and DS: methodology. XH: software. RW: supervision and validation. QZ and HL: writing—review and editing. QZ: conceptualization. HL: team management and coordination. All authors contributed to the article and approved the submitted version.

## Conflict of Interest

The authors declare that the research was conducted in the absence of any commercial or financial relationships that could be construed as a potential conflict of interest.

## Publisher's Note

All claims expressed in this article are solely those of the authors and do not necessarily represent those of their affiliated organizations, or those of the publisher, the editors and the reviewers. Any product that may be evaluated in this article, or claim that may be made by its manufacturer, is not guaranteed or endorsed by the publisher.

## Abbreviations

DS: dietary supplement
RCT: randomized controlled trial
SMD: standard mean difference
SWD: shift work disorder.

## References

[1] American Psychiatric Association. Diagnostic and Statistical Manual of Mental Disorders: DSM-5. Washington, DC: American Psychiatric Association (2013).

[2] Wickwire EM Geiger-Brown J Scharf SM Drake CL. Shift work and shift work sleep disorder: clinical and organizational perspectives. Chest. (2017) 151:1156–72. 10.1016/j.chest.2016.12.007 28012806 PMC6859247

[3] Wang N Sun Y Zhang H Wang B Chen C Wang Y . Long-term night shift work is associated with the risk of atrial fibrillation and coronary heart disease. Eur Heart J. (2021) 42:4180–8. 10.1093/eurheartj/ehab505 34374755

[4] Li M Huang JT Tan Y Yang BP Tang ZY. Shift work and risk of stroke: a meta-analysis. Int J Cardiol. (2016) 214:370–3. 10.1016/j.ijcard.2016.03.052 27085131

[5] Li W Chen Z Ruan W Yi G Wang D Lu Z. A meta-analysis of cohort studies including dose-response relationship between shift work and the risk of diabetes mellitus. Eur J Epidemiol. (2019) 34:1013–24. 10.1007/s10654-019-00561-y 31512118

[6] Cheng WJ Puttonen S Vanttola P Koskinen A Kivimaki M Harma M. Association of shift work with mood disorders and sleep problems according to chronotype: a 17-year cohort study. Chronobiol Int. (2021) 38:518–25. 10.1080/07420528.2021.1885431 33588657

[7] Travis RC Balkwill A Fensom GK Appleby PN Reeves GK Wang XS . Night shift work and breast cancer incidence: three prospective studies and meta-analysis of published studies. J Natl Cancer Inst. (2016) 108:djw169. 10.1093/jnci/djw169 27758828 PMC5241898

[8] Stangherlin A Watson JL Wong DCS Barbiero S Zeng A Seinkmane E . Compensatory ion transport buffers daily protein rhythms to regulate osmotic balance and cellular physiology. Nat Commun. (2021) 12:6035. 10.1038/s41467-021-26725-7 34654800 PMC8520019

[9] Giorgi F Mattei A Notarnicola I Petrucci C Lancia L. Can sleep quality and burnout affect the job performance of shift-work nurses? A hospital cross-sectional study. J Adv Nurs. (2018) 74:698–708. 10.1111/jan.13484 29164664

[10] Lee ML Howard ME Horrey WJ Liang Y Anderson C Shreeve MS . High risk of near-crash driving events following night-shift work. Proc Natl Acad Sci USA. (2016) 113:176–81. 10.1073/pnas.1510383112 26699470 PMC4711869

[11] Cai C Vandermeer B Khurana R Nerenberg K Featherstone R Sebastianski M . The impact of occupational shift work and working hours during pregnancy on health outcomes: a systematic review and meta-analysis. Am J Obstet Gynecol. (2019) 221:563–76. 10.1016/j.ajog.2019.06.051 31276631

[12] Liira J Verbeek JH Costa G Driscoll TR Sallinen M Isotalo LK . Pharmacological interventions for sleepiness and sleep disturbances caused by shift work. Cochrane Database Syst Rev. (2014) 2014:CD009776. 10.1002/14651858.CD009776.pub2 25113164 PMC10025070

[13] Slanger TE Gross JV Pinger A Morfeld P Bellinger M Duhme AL . Person-directed, non-pharmacological interventions for sleepiness at work and sleep disturbances caused by shift work. Cochrane Database Syst Rev. (2016) 2016:CD010641. 10.1002/14651858.CD010641.pub2 27549931 PMC8406755

[14] Chan V Lo K. Efficacy of dietary supplements on improving sleep quality: a systematic review and meta-analysis. Postgrad Med J. (2021) 98:285–93. 10.1136/postgradmedj-2020-139319 33441476

[15] Pereira H Feher G Tibold A Costa V Monteiro S Esgalhado G. Mediating effect of burnout on the association between work-related quality of life and mental health symptoms. Brain Sci. (2021) 11:813. 10.3390/brainsci11060813 34205291 PMC8235172

[16] Page MJ McKenzie JE Bossuyt PM Boutron I Hoffmann TC Mulrow CD . The PRISMA 2020 statement: an updated guideline for reporting systematic reviews. BMJ. (2021) 372:n71. 10.1136/bmj.n71 33782057 PMC8005924

[17] Higgins JP Altman DG Gotzsche PC Juni P Moher D Oxman AD . The Cochrane Collaboration's tool for assessing risk of bias in randomised trials. BMJ. (2011) 343:d5928. 10.1136/bmj.d5928 22008217 PMC3196245

[18] Higgins JP Thompson S Deeks J Altman D. Measuring inconsistency in meta-analyses. BMJ. (2003) 327:557–60. 10.1136/bmj.327.7414.557 12958120 PMC192859

[19] Baradari AG Alipour A Mahdavi A Sharifi H Nouraei SM Emami Zeydi A. The effect of zinc supplementation on sleep quality of ICU nurses: a double blinded randomized controlled trial. Workplace Health Saf. (2017) 66:191–200. 10.1177/2165079917734880 29241421

[20] Bjorvatn B Stangenes K Øyane N Forberg K Lowden A Holsten F . Randomized placebo-controlled field study of the effects of bright light and melatonin in adaptation to night work. Scand J Work Environ Health. (2007) 33:204–14. 10.5271/sjweh.1129 17572830

[21] Cavallo A Ris MD Succop P Jaskiewicz J. Melatonin treatment of pediatric residents for adaptation to night shift work. Ambul Pediatr. (2005) 5:172–7. 10.1367/A04-124R.1 15913411

[22] Farahmand S Vafaeian M Vahidi E Abdollahi A Bagheri-Hariri S Dehpour AR. Comparison of exogenous melatonin versus placebo on sleep efficiency in emergency medicine residents working night shifts: a randomized trial. World J Emerg Med. (2018) 9:282–7. 10.5847/wjem.j.1920-8642.2018.04.008 30181797 PMC6117540

[23] Folkard S Arendt J Clark M. Can melatonin improve shift workers' tolerance of the night shift? Some preliminary findings. Chronobiol Int. (1993) 10:315–20. 10.3109/07420529309064485 8261530

[24] Sadeghniiat-Haghighi K Aminian O Pouryaghoub G Yazdi Z. Efficacy and hypnotic effects of melatonin in shift-work nurses: double-blind, placebo-controlled crossover trial. J Circadian Rhythms. (2008) 6:10. 10.1186/1740-3391-6-10 18957133 PMC2584099

[25] Huffmyer JL Kleiman AM Moncrief M Scalzo DC Cox DJ Nemergut EC. Impact of caffeine ingestion on the driving performance of anesthesiology residents after 6 consecutive overnight work shifts. Anesth Analg. (2020) 130:66–75. 10.1213/ANE.0000000000004252 31274603

[26] Khajehnasiri F Mortazavi SB Allameh A Akhondzadeh S. Effect of omega-3 and ascorbic acid on inflammation markers in depressed shift workers in Shahid Tondgoyan Oil Refinery, Iran: a randomized double-blind placebo-controlled study. J Clin Biochem Nutr. (2013) 53:36–40. 10.3164/jcbn.12-98 23874068 PMC3705155

[27] Smith-Ryan AE Mock MG Trexler ET Hirsch KR Blue MNM. Influence of a multistrain probiotic on body composition and mood in female occupational shift workers. Appl Physiol Nutr Metab. (2019) 44:765–73. 10.1139/apnm-2018-0645 30566363

[28] Thottakam B Webster NR Allen L Columb MO Galley HF. Melatonin is a feasible, safe, and acceptable intervention in doctors and nurses working nightshifts: the MIDNIGHT trial. Front Psychiatry. (2020) 11:872. 10.3389/fpsyt.2020.00872 33192634 PMC7481467

[29] West NP Hughes L Ramsey R Zhang P Martoni CJ Leyer GJ . Probiotics, anticipation stress, and the acute immune response to night shift. Front Immunol. (2020) 11:599547. 10.3389/fimmu.2020.599547 33584665 PMC7877220

[30] Zhang L Zhang R Shen Y Qiao S Hui Z Chen J. Shimian granules improve sleep, mood and performance of shift nurses in association changes in melatonin and cytokine biomarkers: a randomized, double-blind, placebo-controlled pilot study. Chronobiol Int. (2020) 37:592–605. 10.1080/07420528.2020.1730880 32079428

[31] Ker K Edwards PJ Felix LM Blackhall K Roberts I. Caffeine for the prevention of injuries and errors in shift workers. Cochrane Database Syst Rev. (2010) 2010:CD008508. 10.1002/14651858.CD008508 20464765 PMC4160007

[32] Brown JP Martin D Nagaria Z Verceles AC Jobe SL Wickwire EM. Mental health consequences of shift work: an updated review. Curr Psychiatry Rep. (2020) 22:7. 10.1007/s11920-020-1131-z 31955278

[33] Zhao M Tuo H Wang S Zhao L. The effects of dietary nutrition on sleep and sleep disorders. Mediators Inflamm. (2020) 2020:3142874. 10.1155/2020/3142874 32684833 PMC7334763

[34] Ho YT Tsai YC Kuo TBJ Yang CCH. Effects of *Lactobacillus plantarum* PS128 on depressive symptoms and sleep quality in self-reported insomniacs: a randomized, double-blind, placebo-controlled pilot trial. Nutrients. (2021) 13:2820. 10.3390/nu13082820 34444980 PMC8402034

[35] Archontogeorgis K Nena E Papanas N Steiropoulos P. The role of vitamin D in obstructive sleep apnoea syndrome. Breathe. (2018) 14:206–15. 10.1183/20734735.000618 30186518 PMC6118887

[36] Liu L Liu C Wang Y Wang P Li Y Li B. Herbal medicine for anxiety, depression and insomnia. Curr Neuropharmacol. (2015) 13:481–93. 10.2174/1570159X1304150831122734 26412068 PMC4790408

[37] Savarese M Di Perri MC. Excessive sleepiness in shift work disorder: a narrative review of the last 5 years. Sleep Breath. (2020) 24:297–310. 10.1007/s11325-019-01925-0 31471831

[38] Zeng Z Mishuk AU Qian J. Safety of dietary supplements use among patients with cancer: a systematic review. Crit Rev Oncol Hematol. (2020) 152:103013. 10.1016/j.critrevonc.2020.103013 32570150

