# Supplementary figures and images for: Efficacy of Dietary Supplements on Sleep Quality and Daytime Function of Shift Workers: A Systematic Review and Meta-Analysis
Source: Front Nutr. 2022 Apr 28;9:850417. doi: 10.3389/fnut.2022.850417 (PMC9097162; doi:10.3389/fnut.2022.850417)

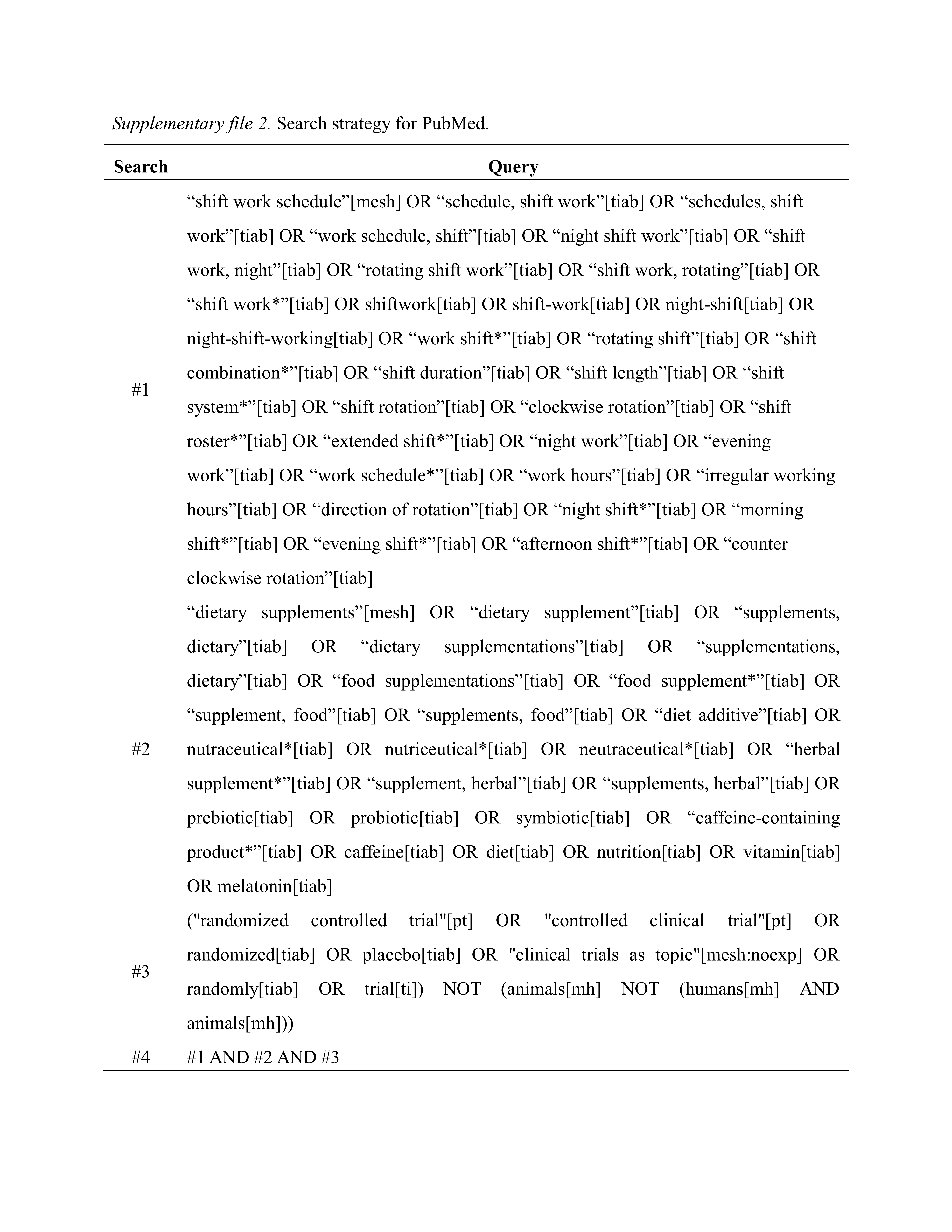

Supplement: Supplementary file 2 [file Image_2.tif]

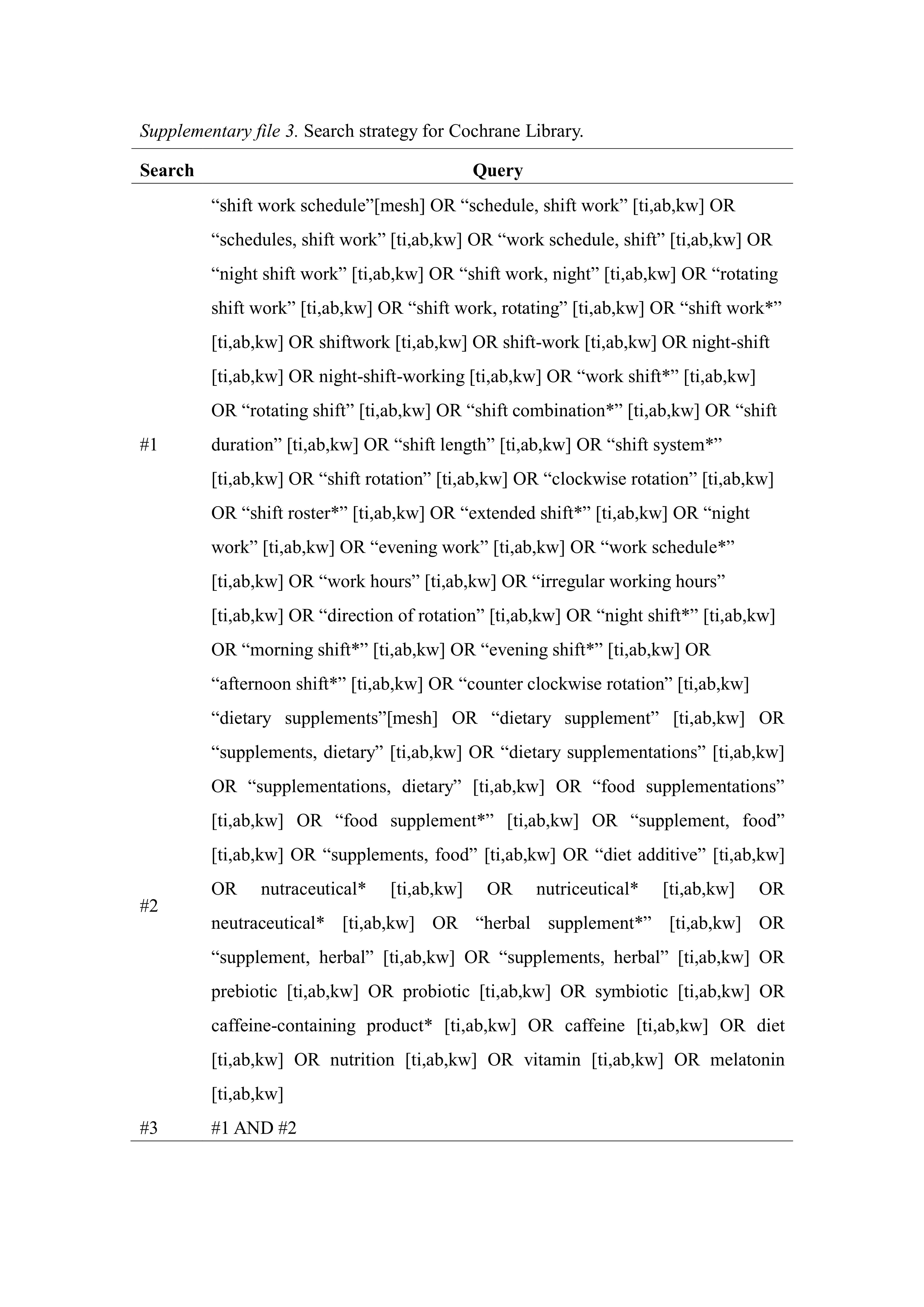

Supplement: Supplementary file 3 [file Image_3.tif]

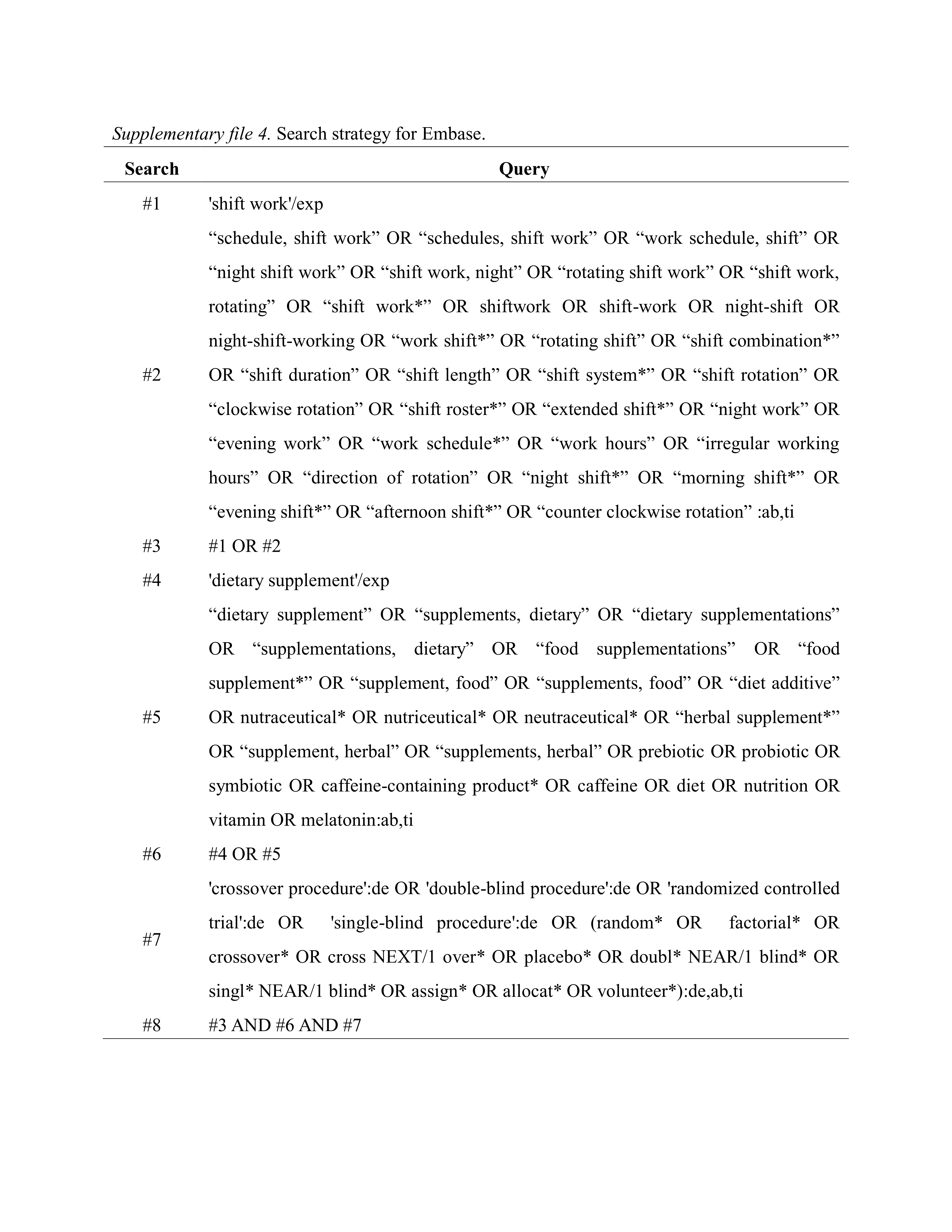

Supplement: Supplementary file 4 [file Image_4.tif]

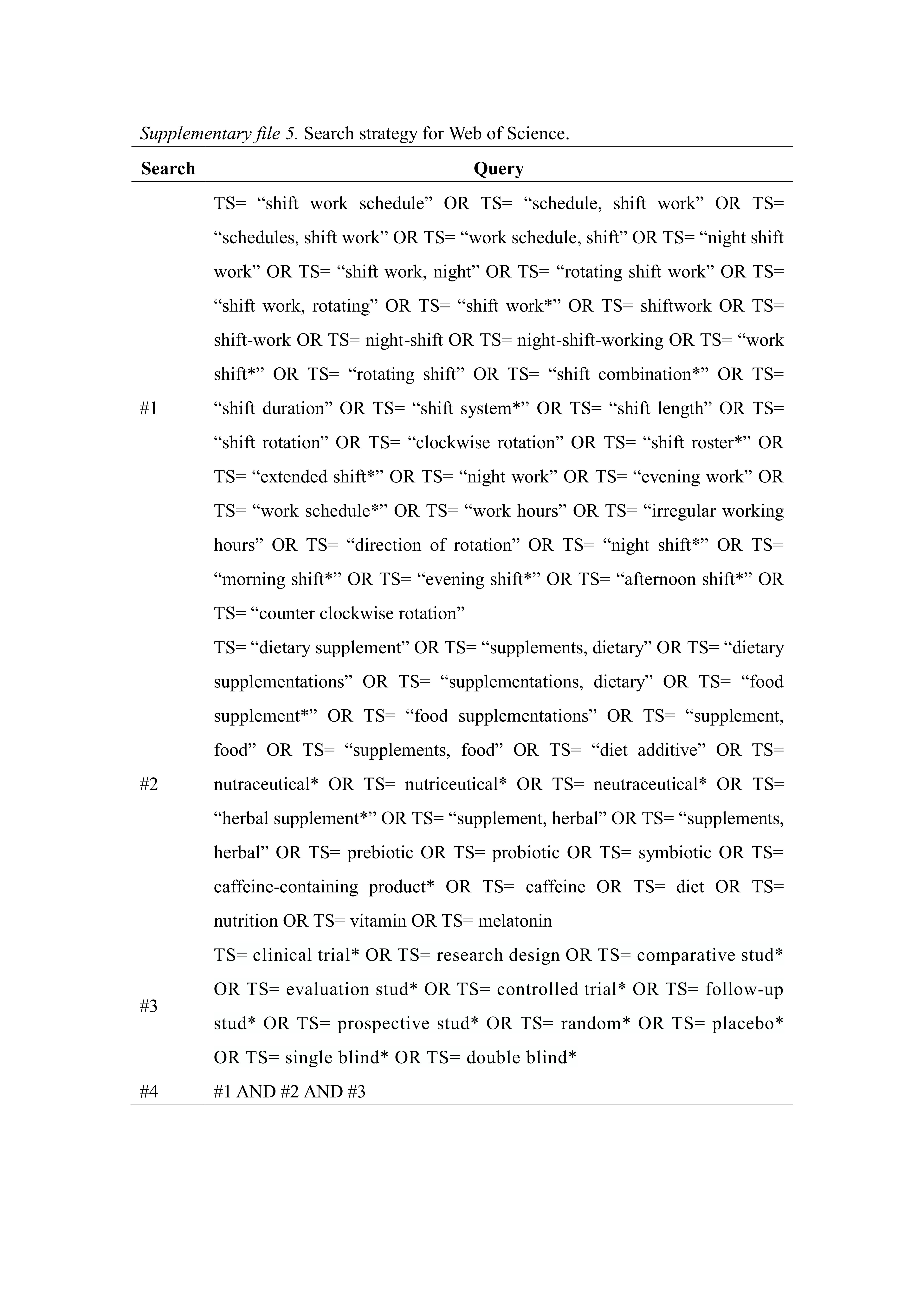

Supplement: Supplementary file 5 [file Image_5.tif]

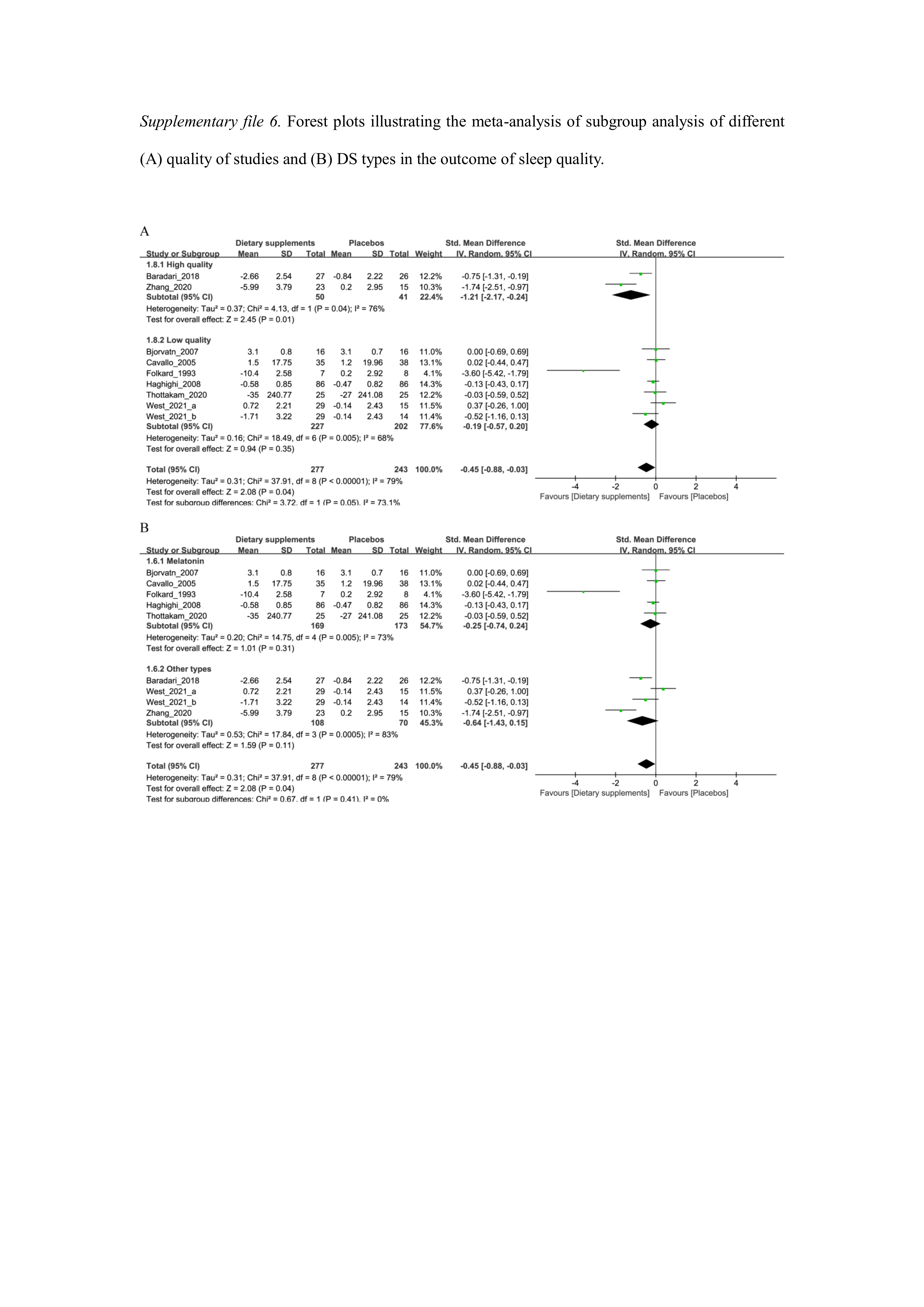

Supplement: Supplementary file 6 [file Image_6.tif]

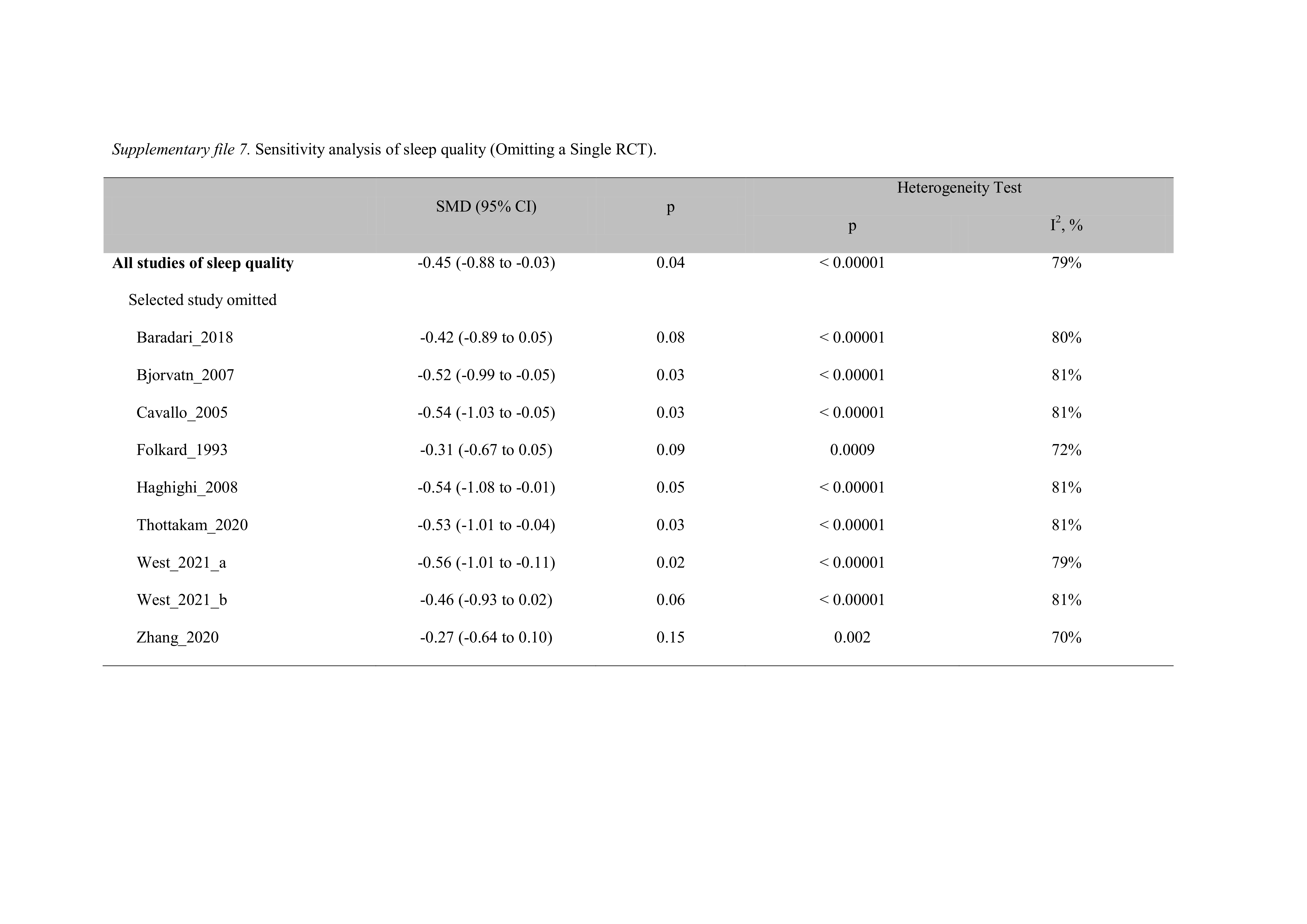

Supplement: Supplementary file 7 [file Image_7.tif]
